# Expression and Role of Heparan Sulfated Proteoglycans in Pancreatic Cancer

**DOI:** 10.3389/fonc.2021.695858

**Published:** 2021-06-25

**Authors:** Simone Furini, Chiara Falciani

**Affiliations:** Department of Medical Biotechnologies, University of Siena, Siena, Italy

**Keywords:** heparan sulfated proteoglycans, pancreatic cancer, prognosis, cancer genomics, screening, precision medicine

## Abstract

Pancreatic cancer is a lethal condition with poor outcomes and an increasing incidence. The unfavourable prognosis is due to the lack of early symptoms and consequent late diagnosis. An effective method for the early diagnosis of pancreatic cancer is therefore sought by many researchers in the field. Heparan sulfated proteoglycan-related genes are often expressed differently in tumors than in normal tissues. Alteration of the tumor microenvironment is correlated with the ability of heparan sulfated proteoglycans to bind cytokines and growth factors and eventually to influence tumor progression. Here we discuss the importance of glypicans, syndecans, perlecan and extracellular matrix modifying enzymes, such as heparanases and sulfatases, as potential diagnostics in pancreatic cancer. We also ran an analysis on a multidimensional cancer genomics database for heparan sulfated proteoglycan-related genes, and report altered expression of some of them.

## Introduction

Pancreatic cancer is a lethal condition with poor outcomes and an increasing incidence. It has a 5‐year survival of 9% (1), the poor prognosis being due to absence of early symptoms and consequent late diagnosis. An effective method for the early diagnosis of pancreatic cancer is therefore sought by many researchers in the field (2, 3).

**Figure 1:**
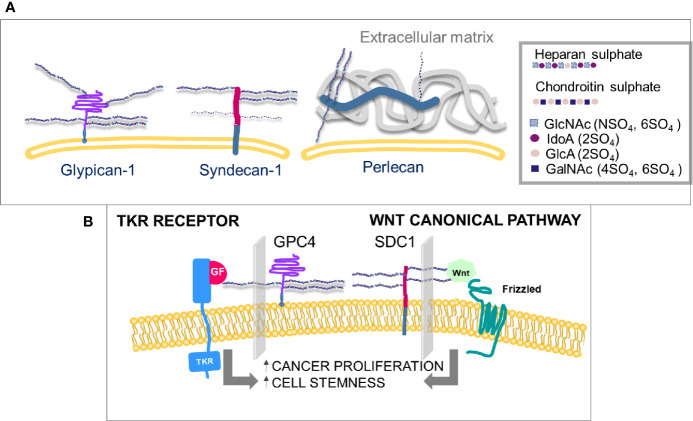
**(A)** Membrane-linked HS-proteoglycans. HS chains in glypicans may can be from three to six and in syndecans from three to five, the latter also carry CS chains, in a maximum of three. The sulfation level of heparin sulfate is higher than any other polysaccharide chain on the cell surface. **(B)** Proliferation and differentiation of cells can be mediated by two independent pathways, growth factors’ receptor activation and canonical Wnt pathways, both involving interactions with specific HS chains. (GlcNAc, N-acetyl glucosamine; IdoA, iduronic acid; GlcA, glucuronic acid; GalNAc, N-acetyl galactosamine; SO4, sulfate group; GF, growth factor; TKR, tyrosine kinase receptor; GPC4, glypican-4; SDC1, syndecan-1).

Heparan sulfate proteoglycans (HSPGs) are prevalently associated with the cell surface or the pericellular matrix. Syndecans and glypicans are transmembrane while perlecan, agrin, and collagen type XVIII are directly secreted in the extra cellular matrix (ECM) once synthesized (4). Heparan sulfate (HS) chains are linear and composed of repeated disaccharide units that alternate amino sugars with glucuronic acid or its epimer iduronic acid (**Figure 1A**). The synthesis and sulfation of sugar chains occur in the Golgi apparatus. The core protein coming from the endoplasmic reticulum is attached to a first tetrasaccharide on a serine residue, then a family of enzymes, exostosins (EXT), carry on the polymerization. The final HS chain length can vary from 50 to 150 disaccharides in length. NDST (N-deacetylase/N-sulfotransferase), HS2ST (Heparan sulfate 2-O-sulfotransferase), HS3ST (Heparan sulfate 3-O-sulfotransferase), HS6ST (Heparan sulfate 6-O-sulfotransferase) enzymes sulfate saccharide residues in positions 2, 3 and 6 (5).

The HS chains of HSPGs bind cytokines and growth factors, generating gradients that control the development of healthy and cancer cells. HSPGs regulate a number of important processes, including cell adhesion, cell migration, endocytosis and exosome formation.

In tumors, HSPGs are often expressed differently than in normal tissues. Alteration of HSPG expression in the tumor microenvironment may result in structural and functional changes that influence tumor progression (6, 7). HSPGs are important in regulating cancer-associated fibroblasts (CAFs) (8) and modulating the effects of cytokines and growth factors on infiltrating cells, such as macrophages and epithelial cells of new blood vessels. Key events, such as tumor angiogenesis (9), cell movement and communication (10) and endocytosis (11) are regulated by HSPGs, making them promising targets for the development of early diagnosis, prognosis prediction and treatment.

The specificity of HSPGs towards cytokines and growth factors is also regulated by extracellular enzymes that transform and modulate the affinity of the binding sites of HSPGs. In FGF2 (Fibroblast growth factor 2) binding to its receptor, FGFR, HS chains of glypican-4 (GPC4) modulate the binding (**Figure 1B**). In canonical Wnt signaling, Wnt binding to its receptor Frizzled is mediated by syndecans-1 (SDC1) (**Figure 1B**). The specific HS chain sulfation profiles are modulated by enzymes, to regulate growth factor and Wnt affinity for their receptors (5). Among such enzymes, sulfatases are considered crucial in the Wnt and growth factors signaling pathways (5, 6) (**Figure 1B**). Sulfatase-1 (SULF1) and Sulfatase-1 (SULF2) hydrolyze the sulfate group in position 6 of heparan sulfates without affecting sulfates in other positions. O-6-sulfation has been demonstrated to be crucial in the signaling pathways of Wnt (12), vascular endothelial growth factor (VEGF), FGF (13) and hepatocyte growth factor (HGF) (14), often with opposite effects. SULF1 and SULF2 are dysregulated in a number of tumors (15), but their role in cancer progression is still controversial.

HSPGs can be detected in human blood through three effects: i) shedding of proteoglycans from cell membranes by the action of sheddases, such as various Matrix Metalloproteinases (MMPs), MMP2, MMP7, MMP9 (16) and ADAM17 (Disintegrin and metalloproteinase domain-containing protein 17) (17); ii) release of HSPG-bearing exosomes into the bloodstream (18, 19); iii) release of polysaccharide chains by the action of heparanases (20). This is why HSPGs have been spotted as potential screening diagnostics in oncology.

In this review we analyzed the role of different HSPGs and extracellular matrix modifying enzymes, as potential diagnostics in pancreatic cancer. We also describe an analysis on a multidimensional cancer genomics database for HSPG-related genes, and report altered expression of some of them in pancreatic cancer.

## The Role of Glypicans in Pancreatic Cancer

Like syndecans and betaglycan, glypicans are HSPGs located on the cell surface (20). Glypicans are anchored to the cell membrane *via* a C-terminal glycosylphosphatidylinositol-(GPI-) moiety and are modified with HS chains near the juxtamembrane region. Six glypicans have been identified in mammals.

### Glypican-1

Glypican-1 (GPC1) has been considered a pancreatic tumor marker for more than 20 years (21). It has been shown to be more strongly expressed by cancer cells and adjacent fibroblasts in human pancreatic cancer than in normal and chronic pancreatitis pancreas tissue. Expression of glypicans in PANC-1 pancreatic cancer cells showed that GPC1was the most expressed, followed by GPC4 and GPC6, while GPC2 was not detectable (22). GPC1 is already known for its ability to regulate signaling of FGF, HGF, TGF-β (Transforming growth factor beta), Wnt and Hedgehog. It is therefore crucial for efficient growth, metastasis and angiogenesis in cancer cells (23). From these important observations, GPC1 has been considered druggable as a marker and/or a targeted drug for pancreatic cancer. In facts, GPC1-targeted and gemcitabine-loaded liposomes efficiently reduced tumor burden in a orthotopic pancreatic cancer (PDAC) mice (24). Besides, an anti-GPC1 antibody conjugated with the cytotoxic agent monomethyl auristatin F, showed significant tumour growth inhibition against GPC1-positive pancreatic cell lines *in vitro* and in a mice xenograft obtained with patient derived-pancreatic cancer cells (25).

GPC1 overexpression was described by Lu H. et al. (26) in a study of 178 pancreatic ductal adenocarcinoma (PDAC) patients from The Cancer Genome Atlas (TCGA) program and 186 subjects whose tissues were used in immunohistochemical staining assays. GPC1 mRNA and the GPC1 protein were scarcely expressed in the normal noncancerous pancreas, whereas in tumor tissues, 59.7% of samples showed positive expression. High levels of GPC1 were associated with poorer differentiation and larger tumor diameters. The authors also observed that expression of the GPC1 gene in cancer tissue could be partly explained by hypomethylation and gene amplification (26).

A few studies stemmed from the finding that GPC1 identifies cancer exosomes and detects early pancreatic cancer (27, 28). Exosomes are extracellular vesicles, containing nucleic acids and proteins, secreted by cells into the extracellular space. They are a particular form of extracellular vesicle, 30–50 nm in diameter, that mediates cell-to-cell signaling. When exosomes are secreted by cancer cells, they can participate in cell–cell communication and tumor progression. Exosomes of different cell origin can be identified and detected by their specific protein enrichment, thus markers from tumor cells on exosomes are very promising for early detection and monitoring of cancer. Melo SA et al. (27) isolated circulating exosomes obtained from mice and from pancreatic adenocarcinomas of patients. These exosomes were enriched in GPC1 and carried mutant Kras mRNA. Exosomes from patients (PDAC, n=190) showed higher expression of GPC1 than did those from healthy donors (n=100), and the higher expression was correlated with higher tumor burden and lower survival. Buscail E et al. (29), conducted a clinical study on 22 patients, to evaluate circulating tumor cells and GPC1+ exosomes as diagnostic tools. Multiple markers carry higher diagnostic value with respect to single ones and facilitates the identification of patients at risk of early disease relapses or fatal outcomes.More generally, pancreas exosomes have raised great interest by virtue of their activity in immune responses (19). The PDAC cell line induces macrophages to develop an M2 anti-inflammatory and pro-tumour phenotype, and therefore increases lymph node metastases and aggravates patient prognosis (30). Costa-Silva et al. (31) also showed that that exosomes from PDAC cells can manipulate distant macrophages, creating a pre-metastatic niche in the liver.

### GPC1 in the Development of Theranostics

On the basis of this knowledge, researchers began to translate the concept of GPC1 as a cancer marker into tools for diagnosis and treatment. Gold nanoparticles have been decorated with an anti-glypican-1-antibody and used for specific detection of pancreatic cancer. The specificity of Gd-Au-NC-GPC1 nanoparticles was studied in an *in vitro* model, comparing uptake by pancreatic cancer (PC) cells (COLO-357-pancreatic adenosquamous carcinoma) and normal cells (human embryonic kidney 293T), and also in an animal model where xenografted PC cells were detected by dual-modal fluorescence imaging/magnetic resonance imaging (FI/MRI) 30 minutes after injection (32).

Similarly, an anti-GPC1-antibody was used to decorate gold nanoparticles which also carried a natural antibacterial, anti-inflammatory, anti-tumor tetracycline diterpenoid compound, oridonin, as well as gadolinium (Gd) and Cy7 dye. The multimodal theranostic nanoparticles (NPs) showed good biocompatibility, as well as potential for multimodal imaging, controllable drug release and effective treatment against tumors, with few side effects (33) in a mouse model of pancreatic cancer obtained with BXPC-3-GFP cells.

A third example with gold nanoparticles, that exploits the pancreas tumor targeting activity of GPC1, uses gold nanosystems for near-infrared fluorescence (NIRF)/MRI, loaded with gemcitabine: GPC1-GEM-NPs. Treatment of xenografted mice with GPC1-GEM-NPs showed a higher tumor inhibitory effect than in controls (34).

Although GPC1 has been used in clinical applications for the early diagnosis of pancreatic cancer, its efficacy as a diagnostic and prognostic tool is still debated. In a study on the exosomes of pancreatic cancer patients (n=6), GPC1 was not diagnostic and its levels remained unvaried 24h after surgical resection (35).

Other researchers observed that GPC1 is also elevated in exosomes of pancreatic cysts, and in other tumors, such as colon cancer (36), and is therefore not strictly specific to pancreatic tumors (37). This lack of specificity for tumor type does not in principle limit the use of GPC1-exosomes as an early pre-diagnostic, also suitable for screening for patients warranting further investigation.

In a study involving 156 patients with PDAC, 199 non‐cancer controls and 240 patients with other cancers, GPC1 and CA19‐9 (carbohydrate antigen 19-9) levels were measured in ELISA. CA19‐9 is considered the gold‐standard for diagnosis of PDAC, but is known to have variable sensitivity and specificity (38). High serum levels of GPC1 predicted poor prognosis in PDAC patients, indeed the overall survival rate 5 years after surgery was shorter in patients with high serum levels of GPC1 than in those with low levels (39). On the other hand, CA19‐9 proved to be significantly better than serum GPC1 as a diagnostic tool, according to Receiver Operating Characteristic curve analysis. The sensitivity and specificity of serum GPC1 were in fact lower than were those of serum CA19‐9. The conclusion of the study was that GPC1 can be considered a good prognostic but not diagnostic tool. The authors also tested the two markers combined (serum GPC1 and CA19‐9), finding higher sensitivity than for the two markers used separately, but the specificity decreased with respect to CA19‐9 used alone.

So far, two main criticisms to the use of GPC1 are easily identified: the lack of a stable standard detection and identification method and the focus on a single marker. The combination of more than one diagnostic probe is a more promising strategy, already followed by other researchers. As an example, Xiao D et al. (40) selected exosomal GPC1, CD82 and serum CA19-9 as multiplex targets. Blood exosomes from patients with pancreatic cancer (n=24), pancreatitis (n=29) and other cancers (n=20) were analyzed by flow cytometry for exosomal GPC1 and CD82 and serum CA19-9. The combination gave excellent diagnostic results, distinguishing patients with pancreatic cancer from those with pancreatitis and healthy subjects.

### Glypican-4

The expression of glypican-4 (GPC4) is significantly upregulated in pancreatic cancer tissue compared with normal tissues and is remarkably correlated with overall survival, according to data from The Cancer Genome Atlas (TCGA) database (41).

GPC4 is described as being involved in the 5-fluorouracil (5-FU) chemoresistance and stem cell–like properties of cancer cells (42). The authors conducted a comprehensive bioinformatic analysis: initially using microarray data derived from the Gene Expression Omnibus (GEO) database, they observed that 5‐FU resistance of pancreatic cancer cells was based on stem-cell–like properties. After identifying the 75 key candidate genes contributing to drug-resistance, their enrichment analysis showed the pathways involved, of which the GPC4 pathway was the most significantly enriched. Furthermore, they showed that knockdown of GPC4 also remarkably attenuated the invasive capacity of pancreatic cancer cells, suggesting that GPC4 might be an upstream regulator of pancreatic cancer stemness (42).

### Glypican-5

Glypican-5 (GPC5) is considered a cancer-related proteoglycan in various cancers (43–46), as tumor suppressor. It was recently observed that mRNA and protein expression levels of GPC5 were significantly down-regulated in PDAC cell lines, including AsPC-1, Panc-1, BxPC-3 and SW1990, compared to the normal pancreatic ductal epithelial cell line (47). Besides, overexpression of GPC5, obtained by transfection, determined a major reduction in AsPC-1 and BxPC-3 cell proliferation.

**Figure 2:**
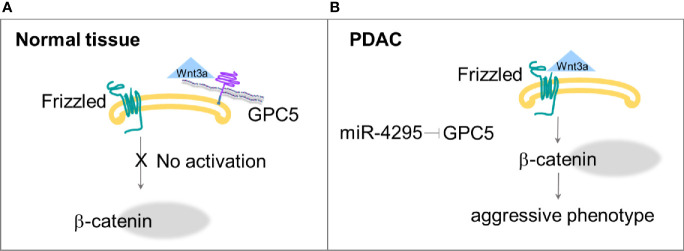
**(A)** GPC5 competes with Frizzled for the binding with Wnt3a, thus Wnt/β−catenin pathway is not activated. **(B)** Down regulation of GPC5 by miR-4295 determines increased activation of Wnt signaling that ends in more aggressive phenotype (45).

These results indicate that the low expression of GPC5 may contribute to PDAC development, which could be explained by the concomitantly reduced expression of catenin and the reduced transcriptional activity of Wnt/β-catenin signaling (45). GPC5 directly bound to Wnt3a and could compete with Frizzled-8 for binding to Wnt3a, at the cell surface (**Figure 2A**). Normally expressed GPC5 could bind to Wnt3a and subsequently inhibit the Wnt pathway activity. When the GPC5 expression is inhibited, Wnt3a would activate Wnt/β-catenin signaling, and lead to cell proliferation, migration and invasion. The authors also postulated that GPC5 is a putative target gene of miR-4295, an oncogenic miRNA in multiple cancers; indeed, inhibition of miR-4295 promotes expression of GPC5 and exhibits an antitumor effect (45) (**Figure 2B**).

## The Role of Syndecans in Pancreatic Cancer

Syndecans are type I transmembrane proteins that consist of 4 members (**Figure 1**). Expression of syndecans in PANC-1 pancreatic cancer cells, quantified by real-time polymerase chain reaction, is reported to be generally higher than that of glypicans (48). Syndecan-4 (SDC4) was the most expressed in PANC-1, followed by syndecan-3 and syndecan-1. SDC1, SDC2 and SDC3 have been described to have a role in pancreatic cancer (49).

### Syndecan-1

SDC1, also known as CD138, is overexpressed on malignant plasma cells and for 25 years has been used as a primary diagnostic marker for multiple myeloma (50). This knowledge has brought to the development, in 2019, of the clinical immunotherapeutic, indatuximab ravtansine, against relapsing or refractory multiple myeloma (51).

In many human microarray datasets, SDC1 is also reported to be significantly up-regulated in PDAC tissue with respect to normal pancreas tissue. However, survival analysis does not show obvious differences between groups with the high expression and medium/low expression of SDC1. SDC1 may be anchored on the cell membrane or shed as soluble SDC1. This is a crucial feature to consider when evaluating expression of SDC1 in a diagnostic or prognostic perspective (52).

A recent integrative bioinformatic study concerned with gene expression profiling showed that SDC1 is a differentially expressed gene (DEG) (53) in pancreatic cancer: 138 common DEGs were identified, 93 of which were up-regulated and 45 down-regulated in pancreatic carcinoma. Gene ontology and KEGG signaling pathway analysis showed that DEGs were rich in extracellular exosomes, plasma membrane and cell-cell interactions, and were involved in extracellular-matrix, proteoglycans-in-cancer and other pathways. The authors indicated SDC1 as a promising molecular marker and therapeutic or diagnostic target for pancreatic cancer, together with other genes, i.e. MET (proto-oncogene receptor tyrosine kinase Met), MELK (maternal embryonic leucine zipper kinase), THBS1 (thrombospondin 1) and TOP2A (DNA topoisomerase II alpha) (53).

**Figure 3:**
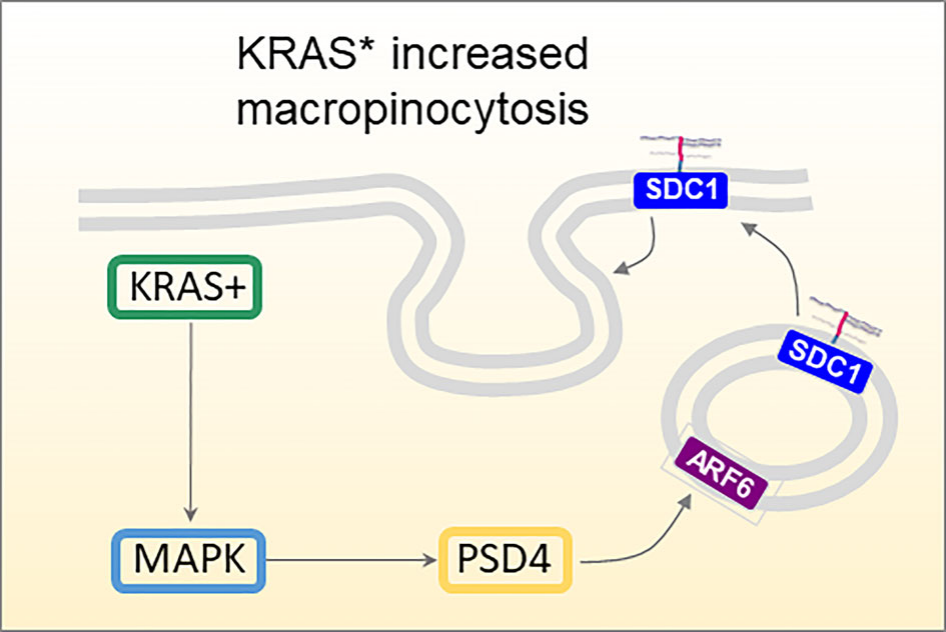
Proposed mechanism of KRAS-MAPK-PSD4-ARF6 driven recycling pathway to mediate SDC1 membrane localization and macropinocytosis in KRAS*-dependent PDAC cells (54).

SDC1 has been studied in association with the oncogene Kras, and especially its constitutively active mutated form Kras^G12D^, Kras*. In iKras* mice, a mouse strain modified genetically to contain inducible Kras* genetic variant, SDC1 expression was observed mostly after oncogene induction by doxycycline treatment and not before, establishing a definite correlation between Kras* and SDC1 expression in PDAC development (54). Furthermore, SDC1 depletion with shRNAs dramatically impaired colony-forming ability, and significantly inhibited tumor growth of subcutaneous xenografts. This was explained by the involvement of MEK (mitogen-activated protein kinase kinase), which once inhibited (with specific inhibitors AZD8330 or trametinib), showed lower SDC1 expression and membrane localization in a dose-dependent manner. The authors also correlated SDC1 and macropinocytosis, an essential metabolic pathway that fuels cell growth in PDAC and other tumors (54, 55), contributing to maintenance and progression of the disease. Kras* pancreas tumor cells showed highly active macropinocytosis, which could be down-regulated by inactivation of Kras* or SDC1. ARF6 (ADP-ribosylation factor 6) and its guanine nucleotide exchange factor PSD4 (PH and SEC7 domain-containing protein 4) are involved in membrane endocytic recycling. The localization of SDC1 on the membrane is controlled by PSD4-ARF6 pathway, which is in turn up-regulated by KRAS* which thus regulates macropinocytosis in PDAC (**Figure 3**).

Yang Y et al. (56) studied SDC1 as a target of the gene expression regulator miR-494 in pancreatic cancer. miR-494 affects the epithelial-mesenchymal transition (EMT) and invasion of pancreatic cancer cells. Pancreatic cancer tissues from 42 patients showed that expression of miR−494 was downregulated and that of SDC1 upregulated. SDC1 was confirmed as the target gene of miR−494. Online target prediction indicated that miR−494 could directly regulate SDC1 and the prediction was confirmed by dual luciferase reporter assay. In this study, cell growth rate, migration, invasion and apoptosis were analysed after transfection with miR−494 mimics and SDC1−siRNA and showed that miR−494 suppressed EMT, migration and invasion of pancreatic cancer cells by inhibiting SDC1 (56).

### Syndecan-2

The significance of SDC2 expression in pancreatic cancer is still debated. SDC2 is reported to be upregulated and correlated with increased transcription of oncogenic proteins involved in migration, such as Kras, Src and ERK2 (57). On the other hand, it has been correlated with patient survival in pancreatic cancer (58).

### Syndecan-3

SDC3 is an essential component for neurite outgrowth. It is expressed in neural tissues and localizes in neuritis (59). Anti-syndecan-3 antibodies inhibited neurite outgrowth *in vitro* (60).

The poor prognosis associated with pancreatic cancer is linked to perineural invasion (61), peritoneal dissemination, and lymph node and liver metastases. Perineural invasion is typical of pancreatic cancer: it is confirmed by histopathology in nearly 100% of cases (62) and is correlated with local recurrence and poor survival. Yao J et al. (63) proved that expression of syndecan-3 and its ligand pleiotropin was significantly associated with perineural invasion.

## HSPG2 (Perlecan)

Pancreatic tumors are known to modify the extracellular matrix and alter interactions between cancer cells and their environment (64). Cancer-associated fibroblasts (CAFs) are found in pancreatic cancer tissues and can promote or restrain disease progression by modifying stroma (65, 66). Perlecan (HSPG2 gene) is present in most pericellular and extracellular matrices, being an essential component of basement membranes. It was recently studied as a key component of the pro-metastatic environment of pancreatic cancer (**Figure 1**). Metastatic CAFs secrete higher levels of perlecan than weakly metastatic cancer. In a cell invasion test, where pancreatic cells invaded a matrix, it was observed that the matrix produced by metastatic CAFs was more readily invaded by pancreatic cancer cells. This effect was promptly reversed when metastatic perlecan knock-out CAFs were used. The observation was confirmed *in vivo* in an orthotopic mouse model, where survival was enhanced and metastatic burden was reduced with metastatic perlecan knock-out CAFs instead of metastatic WT CAFs, using high- and low-metastatic pancreatic cancer cells. Importantly, the authors also showed that pancreatic cancer cells, with mutated p53, triggered a population of CAFs that readily established a pro-metastatic environment (64).

## The Role of Heparanase in Pancreatic Cancer

Heparanases are endoglycosidases which hydrolyze glycoside bonds of GlcA residues at a limited number of specific locations within HS chains and can therefore modulate the glycosidic pattern of the outer side of the cell membrane, playing a crucial role in modulating ligand-binding properties of all HSPGs and in matrix modeling. Heparanase also releases fragments of HS that bind many different growth factors and cytokines and regulate Notch, Hedgehog and Wnt pathways (67). Many cancer types show increased levels of heparanase that promote resistance to chemotherapy, aggressive tumor progression and are therefore associated with poor prognosis (68).

As long as 25 years ago, Lapierre F. et al. (69) reported that a 2,3-O-desulfated heparin derivative, inhibitor of heparanase, significantly reduced the rate of tumor growth of a subcutaneous xenograft obtained with CaPan-2 pancreas adenocarcinoma in nude mice, probably by reducing angiogenesis. Tripathi K. et al. (70) recently observed that high levels of heparanase promote stem-like properties in human myeloma cells and that unlike wild-type cells, cells deficient in heparanase effectively induce tumors in mice. From a mechanistic point of view, the authors also showed that heparanase activated the NF-kB pathway, triggering expression of genes associated with stemness (70). The heparanase inhibitor roneparstat (71) was used to show that inhibiting heparanase activity was crucial for delaying tumor relapse in mice. Roneparstat is an N-acetylated heparin derivative that shows promising antitumor properties and has already undergone a phase I clinical study for treating multiple myeloma (71, 72) as well as being tested against radiation-enhanced pancreatic carcinoma (72, 73). Ionizing radiation was shown to augment the invasive phenotype of pancreatic carcinoma cells *in vitro* and *in vivo* by upregulating heparanase (73). Ionizing radiation treatment of PANC1 pancreatic carcinoma cells determined an increase in Egr1 (Early Growth Response Protein 1), which in turn caused repression of heparanase transcription. The heparanase inhibitor roneparstat was used to demonstrate the role of heparanase in relapse and persistence of pancreatic cancer. Animals bearing an orthotopic model of pancreatic cancer were irradiated and treated with the heparanase inhibitor: tumor growth was significantly reduced with respect to untreated animals. Notably, roneparstat alone did not exert a significant inhibitory effect, probably because the cancer cells used in the study, PANC1, express low levels of heparanase. In a mouse model of pancreatic carcinoma with Panc02, highly expressing heparanase, the heparanase inhibitor showed substantial anti-tumor activity (73).

Necuparanib is a low molecular weight 5.5–6.5 kDa heparin sulfate derivative which has already undergone phase II clinical trial in metastatic pancreas ductal adenocarcinoma (74). The study arose from a previous observation that the invasive behavior of pancreatic tumor cells, co-cultured with stellate cells to mimic pancreatic cancer behavior, could be controlled with necuparanib (75).

PG545 is a fully sulfated tetrasaccharide functionalized with cholestanyl aglycone, that inhibits heparanase and angiogenesis (76). In combination with gemcitabine, PG545 promoted apoptosis and reduced tumor growth in an orthotopic pancreatic tumor mouse model, suppressing Wnt/β-catenin signaling by interacting with Wnt3a and Wnt7a (77, 78).

## The Role of Sulfation Patterns in HSPG in Relation to Pancreatic Cancer

**Figure 4:**
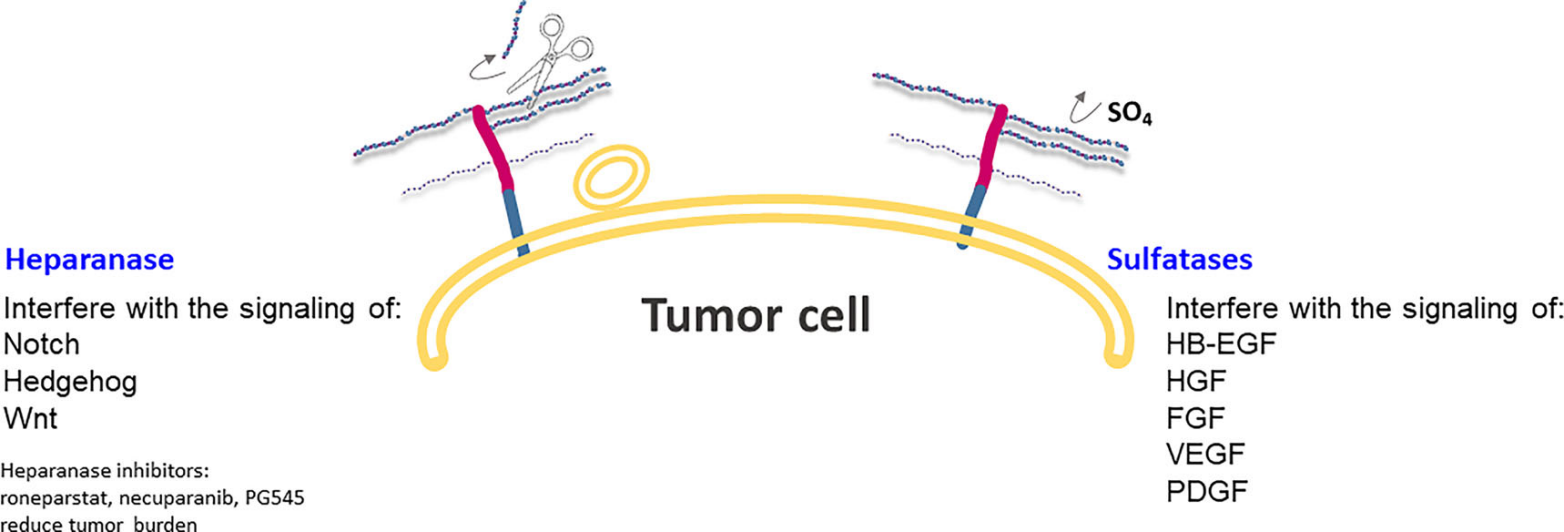
Heparanase and Sulfatases modify HSPGs and tumor microenvironment by altering ligand-receptors interactions and regulating expression of HSPG at the cell surface. Heparanase is also involved in exosome formation and in the change of a stimulus from autocrine to paracrine.

SULF1 and SULF2 are extracellular enzymes that catalyze the hydrolysis of 6-O-sulfation of heparan sulfate, without affecting the N-, 2- or 3-O positions (**Figure 4**). The sulfation state of HSPG glycan chains determines the binding of a large number of signaling ligands and their receptors (79); in fact, HSPGs often act as coreceptors contributing to the activation of specific pathways of cells. More specifically, 6-O sulfation is very significant in the signaling activity of growth factors, such as heparin-binding epidermal growth factor (HB-EGF), hepatocyte growth factor (HGF), fibroblast growth factor (FGF), vascular endothelial growth factor (VEGF) and platelet-derived growth factor (PDGF), as well as Wnt (13, 80). SULF1 and SULF2 act at the interface between tumor cells and their microenvironment, taking part in key interactions between local tumor and host cell interactions (**Figure 4**).

The expression of SULF1 and SULF2 enzymes is differently regulated in a number of tumors, but their precise role in tumor growth has remained controversial. To further complicate the scene, alternative RNA splicing can alter the binding characteristics and enzyme activities of SULF1 and SULF2 (15), finely modulating proliferation and migration features in cancer cells bearing this extracellular enzyme.

As long as 15 years ago, Abiatari et al. (81) documented that SULF1 reduced the growth of PANC-1 pancreatic cancer cells, but at same time increased their invasiveness as measured in an orthotopic mice model of pancreatic cancer. Human samples of surgical resections of pancreatic cancers (n=15) showed strong staining of SULF1, which decreased in metastatic tissue samples, suggesting that complete desulfation of HSPGs occurs mainly in the process of invasion.

Lyu Y et al. (82) analyzed 65 pancreatic cancer specimens and showed that SULF1 expression was higher in pancreatic cancer tissue than in normal tissues. High SULF1 expression was also associated with poorer prognosis, hence more advanced TNM (Tumor, Node, Metastasis) stages, and was correlated with higher CA19-9 levels, despite smaller tumor size.

Elevated expression of SULF2 was detected in PDAC (79), and impairment of SULF2, obtained by silencing or by lentiviral shRNA-mediated inactivation, reduced cancer cell growth *in vitro* and *in vivo* by enhancing the Wnt pathway.

Alhasan SF et al. (83) analyzed 93 samples from patients who underwent PDAC resection. Elevated expression of SULF2 was associated with vascular invasion and advanced tumor stage and shorter overall survival. Briefly, 60% of patients had more than 25%-positive SULF2 cells and the higher level of SULF2 was correlated with tumor stage, perineural invasion and vascular invasion.

## cBioPortal Analysis of HSPG-Related Genes in Pancreatic Cancer

cBioPortal (84, 85) is a resource for interactive exploration of multidimensional cancer genomics data sets, by storing expression data and clinical attributes. We analysed data from four studies: TCGA - PanCancer Atlas (86), QCMG-Nature 2016 (87), ICGC, Nature 2012 (88) and UTSW-Nat Commun 2015 (89), that included a total of 848 patients. We queried alterations in the genes most frequently involved in HSPG expression and post translational modifications, i.e. HSPG2 for perlecan, SDC1, SDC2, SDC3, SDC4 for syndecans, GPC1, GPC2, GPC3, GPC4, GPC5, GPC6 for glypicans, SULF1, SULF2 for sulfatases, HS6ST1, NDST1, NDST2, NDST3, NDST4 for sulfo transferases and HPSE for heparanase. The reply was that all the genes of interest had a certain (low) level of alteration.

The alteration of mRNA expression in the genes of interest, was estimated as a z-score and determined for each sample by comparing mRNA expression with its distribution in a reference population harboring typical expression of the gene. In cBioportal, the expression distribution for unaltered copies of the gene is estimated by calculating the mean and variance of the expression values for samples in which the gene is diploid (i.e. value is “0”). Diploid is regarded as the unaltered distribution.

**Figure 5:**
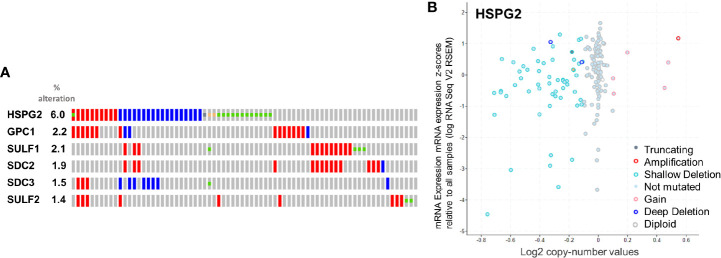
**(A)** rate of alteration of the queried genes. **(B)** Copy-number values (x axis) against mRNA expression z-scores (y axis) relative to all samples for HSPG2.

Before investigating which of the genes were the most altered, within those selected, we looked at their mRNA Expression, as RSEM (RNA-Seq by Expectation Maximization) (90) which quantifies transcripts (**Figure 5**). Genes with less than 100 diploids copies were excluded from further analyses.

The level of expression of syndecans and glypicans was correlated with that of PANC-1 pancreatic cancer cells, quantified by real-time polymerase chain reaction (48). In all four studies, mRNA expression, taken as the median RSEM level of syndecans, was generally higher than that of glypicans. Among syndecans, SDC4 was the most expressed in PANC-1, followed by SDC3 and then SDC1. Among glypicans, GPC1 was the most expressed, followed by GPC4 and GPC6, whereas GPC2 was not detectable (48). In the four studies included in the cBioportal analysis, SDC4 and SDC1 were more highly expressed than SDC3 and SDC2, which were the least expressed. GPC1, GPC4 and GPC6 were all expressed less than syndecans, while GPC2, GPC3 and GPC5 were so poorly represented that they were excluded. Likewise, NDST2, NDST3, NDST4 and HPSE were not considered for evaluation because of their low expression.

The selected genes were evaluated for their rate of alteration. According to the analytical report of cBioportal, HSPG2, GPC1, SULF1, SDC2, SDC3 and SULF2, in that order, were altered more than the others (**Figure 5**). The result is in line with what we previously reported in this review regarding HSPGs in pancreatic cancer, where HSPG2, GPC1, SULF1, SDC2, SDC3 and SULF2 were appointed key players in tumor progression. The absence of SDC1 among the altered genes is at variance with the findings of other authors, also reported above. This inconsistency is most probably due to the fact that none of the studies in the cBioPortal database report protein expression level, which may be nonlinear and particularly mismatched in the case of SDC1.

## Conclusion

Precision medicine for cancer patients is now considered a cutting-edge approach, adopting the most suitable options from diagnosis to treatment of individuals. In other words, the therapeutic indication does not concern the type of pathology but the individual’s manifestation of it. Liquid biopsy, i.e. blood sampling to detect specific markers, helps oncologists to stratify patients for the most suitable treatment, and to monitor treatment response and resistance mechanisms in a timely manner. Circulating markers also help to evaluate the risk of metastatic relapse and to estimate prognosis. Screening campaigns based on minimally invasive low-cost liquid biopsy will enormously improve early diagnosis, and therefore prognosis, and eventually lower the disease burden of society.

Research in this field has already shown that a single marker for a type of cancer is seldom resolutive or decisive; groups of markers are more informative. In this perspective, HSPGs localized in the ECM or on the outer face of the cell membrane and functionally shed are interesting candidates.

Here we outlined the remarkable features of HSPGs, such as GPC1, GPC4, GPC5, SDC1, SDC2, SDC3 and HSPG2, and some HSPG-related proteins like heparanase and SULF1/2, in cancer diagnosis. We explored four multidimensional cancer genomics data sets using cBioPortal applications, querying HSPG-related genes: HSPG2, SDC1, SDC2, SDC3, SDC4, GPC1, GPC2, GPC3, GPC4, GPC5, GPC6, SULF1, SULF2, HS6ST1, NDST1, NDST2, NDST3, NDST4 and HPSE. We discarded genes with a low copy number to avoid overestimating the number of variations and focused on HSPG2, GPC1, SULF1, SDC2, SDC3 and SULF2, which showed the highest levels of mutations in the group, in line with previous findings that designated them as promising markers. The huge contribution of next generation sequencing technologies combined with *in vitro* assays of HSPG-related proteins will generate a strong boost in the field of liquid biopsy for precision medicine remarkably in the next few years.

## Author Contributions

CF conceived the review, collected papers and summarized the data. SF reviewed the bioinformatics data. All authors contributed to the article and approved the submitted version.

## Funding

Funding from Regione Toscana, project Pancreas Early Diagnosis, n D73J20000040002.

## Conflict of Interest

The authors declare that the research was conducted in the absence of any commercial or financial relationships that could be construed as a potential conflict of interest.
